# Supplementary material for: Chromothripsis during telomere crisis is independent of NHEJ, and consistent with a replicative origin
Source: Genome Res. 2019 May;29(5):737–49. doi: 10.1101/gr.240705.118 (PMC6499312; doi:10.1101/gr.240705.118)
Supplement: Supplemental Material [file supp_gr.240705.118_Supplemental_file_1.zip › contigs/annotated_contigs/DB51/contig.2.DB51_length_311_mean_cov_3.75241157556.docx]

**DB51_length_311_mean_cov_3.75241157556**

```
AGGATGGCACAGATGTTTCAAACTACTTAACAG|ATAATTT|TAATTAAGAAATTATCTGTTAAGTAGTTTGAAACATCTGTGCCATCC
 >chr4:70114438-70114478 - E=1e-12 >chr4:70114423-70114701 + E=1e-156
TTTCTTTTCAAATGTATGAATTTTTTTATAAGTTCCCACAAATCAAAATTATGGAAAAAAAAGACACAATCCCAAAGAAAATTTATCAT

AGAACAATGGAACATAGGTAATTCTCTAGCTCATTCGTCTTCAATAAAACAAATAAATATAAGAAGACAAAGGTCAGGAAGGAAATAGA

GAAGAAAAGATAACCGATTATCCAAAACACACACAAAATTGAAAGC
```
